# Editorial: Protein Crops: Food and Feed for the Future

**DOI:** 10.3389/fpls.2017.00105

**Published:** 2017-02-06

**Authors:** Antonio M. De Ron, Francesca Sparvoli, José J. Pueyo, Didier Bazile

**Affiliations:** ^1^Biology of Agrosystems, Misión Biológica de Galicia, Consejo Superior de Investigaciones Científicas (CSIC)Pontevedra, Spain; ^2^Institute of Agricultural Biology and Biotechnology, Consiglio Nazionale delle RicercheMilan, Italy; ^3^Institute of Agricultural Sciences (ICA), Consejo Superior de Investigaciones Científicas (CSIC)Madrid, Spain; ^4^Directorate General in Charge of Research and Strategy, French Agricultural Research and International Cooperation Organization (CIRAD)Montpellier, France; ^5^Research Unit Management of Renewable Resources and the Environment (UPR GREEN) (CIRAD)Montpellier, France

**Keywords:** cereals, legumes, plant proteins, quinoa

Because of the protein content of their seeds, grain legumes, pseudocereals, and other crops are candidates to satisfy the growing demand for plant protein for food and feed. Crop production worldwide is highly specialized and currently relies on a very small number of species, raising questions about the sustainability of farming (Tilman et al., 2002). The role of legumes in nutrition has been recognized as a relevant source of plant protein together with other benefits for health. Soybean, peanut, common bean, pea, lupine, chickpea, faba bean, lentil, grass pea, cowpea, and pigeon pea are currently the most important legumes for human consumption and animal feed (De Ron, 2015). The integration of legumes into agriculture could reduce the current protein deficit and contribute to the transition to more sustainable agricultural systems. Legumes contribute to the sustainable improvement of the environment due to their ability to fix nitrogen and their beneficial effects on the soil (Drevon et al., 2015), having a tremendous potential in the reclamation of poor and marginal lands for agriculture (Coba de la Pe-a and Pueyo, 2012). Other protein crops include some minor crops, such as flax, hemp, or caraway, and some cereals have a certain potential, for they are quite abundant in food and feed. However, most importantly, chenopods called pseudocereals, such as amaranth and quinoa are recognized as excellent sources of protein and their seeds contain in particular the lysine, an essential amino acid that is limited in cereals. Nutritional evaluations of quinoa indicate that it constitutes a source of complete protein with a good balance of all of the amino acids needed for human diet, and also important minerals, vitamins, high quality oils, and flavonoids.

In this Research Topic (Protein crops: Food and feed for the future), were included papers dealing with different aspects of protein crops, such as breeding and selection of varieties for high yield or specific traits, biodiversity, sustainable cultivation, food and feed uses, nutritional value, health benefits, and socio-economic or environmental issues, which may be considered crucial to help provide the plant proteins of the future.

A novel assessment framework was developed by Reckling et al. and applied in five case study regions across Europe with the objective of evaluating trade-offs between economic and environmental effects of integrating legumes into cropping systems. Cropping systems with legumes significantly reduced nitrous oxide emissions and N fertilizer use in arable and forage systems. However, grain legumes reduced gross margins in 3 of 5 regions, while forage legumes increased gross margins in 3 of 3 regions. Legumes have been proved also beneficial in crop rotation and intercropping with cereals. Correa-Galeote et al. report on the variation of bacterial communities in the rhizosphere of maize as a result of intercropping with bur clover.

Therefore, increasing the cultivation of legumes could lead to economic competitive cropping systems and positive environmental impacts, but achieving this aim requires the development of novel management strategies informed by the involvement of advisors and farmers and it is often necessary to support farmers in their efforts to improve the biodiversity level on their farms. Since many legumes are bee-pollinated, the importance of the plant–pollinator interplay (PPI) in legumes lies in a combination of the importance of pollination for the production service and breeding strategies, plus the increasing urgency in mitigating the decline of pollinators through the development and implementation of conservation measures. The study by Suso et al. about PPI encompasses a range of reviews, opinions and perspectives and two basic approaches are proposed: (a) farming with alternative pollinators and (b) crop design system.

Lucas et al. focus on the future of lupin as a protein crop in Europe and conclude that lupin can be capable of promoting socio-economic growth and environmental benefits in Europe, provided that advanced breeding techniques deliver new productive lupin varieties, and novel processes are optimized to obtain high-quality protein ingredients for marketable foods to be offered to consumers. The need for optimizing lupin protein processing for increased nutritional value and food safety is also addressed by Bartkiene et al.

There is a growing awareness of the necessity to consider seed quality traits in breeding programs of protein crops. Bellaloui et al. discuss an agronomic study aimed to analyse the effects of management practices (planting date and seeding rate) on soybean seed composition. Seed storage proteins are a valuable source of essential amino acids for the diet; however in many legume seeds they often possess biological activities that might be either beneficial or detrimental to the consumer. Sparvoli et al. show how genetic removal, in common bean, of lectins and retention of α-amylase inhibitor together with the reduction of phytate accumulation, may prove useful to produce bean composite flours for the preparation of novel foods, biscuits, with improved nutritional properties. Herman and Schmidt discuss on the potential for engineering soybeans for aquaculture feed by removing anti-nutritional proteins and increasing beneficial compounds.

Almost 50% of the global food protein supply comes from cereal seeds (faostat.fao.org). Most of these proteins (prolamins) accumulate in protein bodies (PB), large polymers formed in the endoplasmic reticulum (ER). On the contrary, the most common classes of seed storage proteins, the 2S albumins and the 7S/11S globulins accumulate in protein storage vacuoles (PSVs), which are the first subcellular compartment that evolved to store seed proteins. As the ER is mainly a compartment of transit, the permanent ER residence of proteins that do not play a role in typical ER functions seems a feature unique to prolamins. Based on the presence of specific domains in the different classes of storage proteins, together with phylogenetic studies of storage proteins evolution, Pedrazzini et al. formulate a hypothesis on how the PB evolved from the PSV as a simple solution to accommodate prolamin storage proteins. Li et al. analyse the variation of albumin, globulin, and glutenin contents in wheat grains as related to nitrogen application rates during different growth stages, which could be an effective approach to modulate the distribution of protein fractions for specific end-uses.

Ramalingam et al. review several proteomic and metabolomic studies on model and crop legumes, and discuss how integration of “omics” will contribute to the identification of accurate biomarkers in legume smart breeding programs. They discuss different methodological approaches, together with bioinformatics, and show how these could be used to tackle biological questions for understanding stress response mechanisms, cellular, and developmental processes and symbiosis.

Other protein crops also deserve attention. Hemp is an ancient crop that has been cultivated worldwide until the early twentieth century, after which its cultivation declined. Recently, interest in this multipurpose crop delivering fibers, shives, and seeds, has been renewed by an increasing demand not only for natural fibers but also for the high content and quality of seed protein and oil. Galasso et al. studied the composition of a collection of hemp genotypes of different geographical origins in order to identify potential genotypes to improve hemp cultivars. Their results reveal noticeable differences among hemp seed genotypes for antinutritional components, oil, and protein content. Collectively, this study suggests that the hemp seed is an interesting product in terms of protein, oil and antioxidant molecules, but a reduction of phytic acid would be desirable for both humans and monogastric animals. Bellaloui et al. and Yang et al. investigated the nutritional value of cotton, regarding protein content in seeds and embryos, respectively.

Crops growth is confronted by a variety of pathogens. Remaining healthy depends on their ability to recognize pathogens and to activate defense mechanisms against them. The plant defense responses are regulated by a broad number of signaling pathways. Transcription factors (TFs) control the transfer of genetic information from DNA to RNA by activation or repression of transcription, playing important roles in plant development and defense by regulating different signaling pathways (Singh et al., 2002; Udvardi et al., 2007).

Curto Rubio et al. used high-throughput quantitative real-time PCR (qPCR) technology to screen more than 1000 *Medicago truncatula* TFs in susceptible and resistant genotypes of *M. truncatula* after infection by the pathogen fungus *Erysiphe pisi* (powdery mildew). Seventy-nine TF genes, belonging to 33 families showed a significant transcriptional change in response to *E. pisi* infection. Forty eight TF genes were differentially expressed in the resistant genotypes compared to the susceptible one in response to *E. pisi* infection, including pathogenesis-related transcriptional factors. The results suggest that these TF genes are among the *E. pisi* responsive genes in resistant *M. truncatula* that may constitute a regulatory network which controls the transcriptional changes in defense genes involved in resistance to *E. pisi*.

The common bean is affected by a wide diversity of fungal pathogens, among them *Rhizoctonia solani* is one of the most important. Many *Trichoderma* species are well-known for their ability to promote plant growth and defense. Mayo et al. studied how the interaction of bean plants with *R. solani* and/or *Trichoderma* affect the plants growth and the level of expression of defense-related genes. *Trichoderma* isolates were evaluated *in vitro* for their potential to antagonize *R. solani*. The interaction of plants with *R. solani* and/or *Trichoderma* affects the level of expression of seven defense-related genes. Later on, Mayo et al. propose the use of biocontrol agents (BCAs) as a strategy to control bean diseases, mainly those caused by fungi. They combined *in silico* analysis and real time PCR to detect additional bean defense-related genes, regulated by the presence of *Trichoderma velutinum* and/or *R. solani*. Based in this strategy, from the 48 bean genes initially analyzed, 14 were selected, and only *WRKY33, CH5b*, and *hGS* showed an up-regulatory response in the presence of *T. velutinum*. The strategy described in this work has shown to be effective to detect genes involved in plant defense, which respond to the presence of a BCA or to a pathogen and also to the presence of both.

Common bean is sensitive to low temperatures. Rapid and uniform seed germination and seedling emergence under diverse environmental conditions is a desirable characteristic for crops. Common bean genotypes differ in their low temperature tolerance regarding growth and yield. Cultivars tolerant to low temperature during the germination and emergence stages and carriers of the grain quality standards demanded by consumers are needed for the success of the bean crop. De Ron et al. studied the performance of 28 dry bean genotypes in open field and in growth chamber under low, moderate, and warm temperature. Screening of seedling emergence and phenotypic response of the bean germplasm under a range of temperatures in controlled growth chambers and under field conditions showed several genotypes with low temperature stress-tolerance at emergence and high yield potential that could be valuable genetic material for breeding programs.

Cowpea is a warm season legume that accounts for a huge portion of the dietary protein of the people in sub-Saharan Africa; however it is affected by many virus diseases that reduce yields. Nsa et al. studied the effects in three cowpea cultivars of single infections and co-infections of three unrelated viruses: Cowpea aphid-borne mosaic virus (CABMV), genus *Potyvirus*, cowpea mottle virus (CMeV), genus *Carmovirus*, and Southern bean mosaic virus (SBMV), genus *Sobemovirus*. The treated plants were assessed for susceptibility to the viruses, growth, and yield. In all cases, early inoculation resulted in higher disease severity compared with late infection. Single, double and triple infections by CABMV, CMeV, and SBMV led to a complete loss of seeds in the three cowpea cultivars; only cultivar White produced some seeds at 30 DAP.

Lentil is the third most important cool-season grain legume in the world after chickpea and pea. Khazaei et al. studied the genetic diversity and population structure of lentil germplasm collection of 352 accessions from 54 countries to estimate genetic diversity and genetic structure using 1194 polymorphic single nucleotide polymorphism (SNP) markers which span the lentil genome. Using principal coordinate analysis, population structure analysis and UPGMA cluster analysis, the accessions were categorized into three major groups: (a) South Asia (sub-tropical savannah), (b) Mediterranean, and (c) northern temperate. Based on the results from this study, it is also clear that breeding programs still have considerable genetic diversity to mine within the cultivated lentil.

A number of articles focus on the importance of quinoa as a climate resilient grain option for climate change (Ruiz et al., 2014). This recent international recognition, for an underutilized crop from the Andean highlands, is largely due to its high nutritional value but underlines the importance of its high genetic diversity for adaptation to new environments (Bazile et al., 2015).

Bazile et al. give a critic of trends on the global expansion of quinoa. After centuries of neglect, the potential of quinoa was rediscovered during the second half of the twentieth century, and now more than 100 countries are testing or cultivating quinoa worldwide. Thanks to the high levels of genetic diversity, the crop is highly resilient to agro-ecological extremes and is tolerant to frost, drought, and salinity. The geographical increase in distribution of quinoa has highlighted the difficulty of access to quality seed, which is a key factor for testing the crop outside the Andes. In this context, research partnerships have allowed trials to be undertaken in non-traditional areas of cultivation.

Following the International Year of Quinoa in 2013, the Food and Agriculture Organization of the United Nations (FAO) initiated various trials at global level. Bazile et al. present field evaluations that were conducted in Asia and the Near East and North African countries. In each of the nine countries involved, the trials were carried out in different locations that globally represent the diversity of 19 agrarian systems for comparing 21 genotypes of quinoa using the same experimental protocol across the locations. Some genotypes showed higher yields for some specific locations when other genotypes were evaluated with stable and satisfactory levels of yield in each of the different trial sites. This production stability is of considerable importance especially under climate change uncertainty.

Choukr-Allah et al. consider the high tolerance and resistance of quinoa to many stresses and various cultivars have been screened for tolerance to salinity, water-use efficiency and nutritional quality. The authors summarized 15 years of studies on assessing the potential for introducing the crop in countries of the Middle East and North Africa and Central Asia and describe the key constraints for scaling-up the production under marginal growing conditions. Quinoa maintains productivity in poor soils and under water stress conditions and high salinity. Nevertheless, higher yields do not guarantee quinoa's success in the region and to be successful it must fit in the current cropping patterns, farming systems, marginal lands affected by salinity and alkalinity, and in the areas where the majority of the food crops could not be produced economically.

Also related to salt stress, Aloisi et al. reported the changes in proteomic and amino acid profiles, phenolic content and antioxidant activity in different quinoa cultivars when subjected to salinity stress. Wu et al. address the question of how the fertilization level influences salinity tolerance of quinoa and how soil salinity and fertility impact on seed quality traits, with particular attention to protein content and seed hardness and seed density.

Benlhabib et al. present an analysis of genetic diversity of 72 F_2:6_ recombinant-inbred lines and parents developed through hybridization between highland and coastal germplasm groups of quinoa and evaluated for quantitative and qualitative traits. The study highlighted the extended diversity regenerated among the 72 lines and helped to identify potentially adapted quinoa genotypes for production in the Moroccan coastal environment, in particular with some lines resistant to downy mildew. Stetter et al. present a complementary research to Benlhabib et al. focused on amaranth. It appears of evidence that for the improvement of minor crops, efficient crossing methods are the basis of breeding programs. The authors developed three different crossing methods and compared their efficiency validated with genetic markers. The rapid production of segregating populations makes amaranth an attractive model for improvement by plant breeding.

Murphy et al. highlight the methods used for plant breeding and their impact on biodiversity in agricultural systems. Maintaining and increasing quinoa biodiversity is imperative, as the dynamics of the global expansion of quinoa may constitute a threat to farmers if the spread is generated with a narrow genetic base. Evolutionary participatory breeding (EPB) appears as a useful tool to develop new quinoa genetic material in cooperation with farmers. The global collaborative network on quinoa (GCN-Quinoa) could be the baseline for participatory plant breeding programs to meet the needs of farmers across a diversity of agrosystems.

While these results on quinoa research suggest that this Andean crop is able to grow in many different environments, social, and cultural considerations remain crucial regarding its possible introduction in new cropping systems worldwide.

## Author contributions

AD, FS, JP, and DB: drafting and revising the work and approval of the version to be published.

### Conflict of interest statement

The authors declare that the research was conducted in the absence of any commercial or financial relationships that could be construed as a potential conflict of interest.
